# Long-term surgical outcomes of Ahmed valve implantation in refractory glaucoma according to the type of glaucoma

**DOI:** 10.1186/s12886-022-02493-w

**Published:** 2022-06-20

**Authors:** Yong Koo Kang, Jae Pil Shin, Dai Woo Kim

**Affiliations:** Department of Ophthalmology, School of Medicine, Kyungpook National University, 680 gukchaebosang-ro, Jung-gu, Daegu, 41944 South Korea

**Keywords:** Ahmed glaucoma valve, Intraocular pressure, Refractory glaucoma

## Abstract

**Background:**

We evaluated the long-term efficacy and surgical outcomes of Ahmed glaucoma valve (AGV) implantation in patients with refractory glaucoma by glaucoma type.

**Methods:**

In total, 135 eyes of 135 patients diagnosed with refractory glaucoma and underwent AGV implantation between 2002 and 2018 were reviewed retrospectively. The best-corrected visual acuity (BCVA), intraocular pressure (IOP), and number of antiglaucoma medications were investigated at baseline and follow-up. The cumulative probability of qualified success according to the glaucoma type was evaluated at 12, 24, 36, and 60 months postoperatively.

**Results:**

The mean IOP of all patients was 35.7 ± 11.7 mmHg, which was significantly reduced 12.7 ± 7.0 mmHg at 1 week, 16.2 ± 7.5 mmHg at 2 weeks, 17.6 ± 6.8 mmHg at 1 month, 17.5 ± 6.4 mmHg at 3 months, 16.1 ± 6.0 mmHg at 6 months, 16.7 ± 8.0 mmHg at 12 months, 16.4 ± 6.6 mmHg at 24 months, 15.6 ± 5.0 mmHg at 36 months, and 15.6 ± 5.6 mmHg at 60 months after surgery (*p* < 0.001, respectively). The mean number of antiglaucoma medications was 3.7 ± 1.3, which significantly decreased to 0.4 ± 0.9 at 1 week, 0.3 ± 0.8 at 2 weeks, 0.7 ± 0.9 at 1 month, 1.1 ± 1.1 at 3 months, 1.4 ± 1.0 at 6 months, 1.5 ± 1.1 at 12 months, 1.6 ± 1.2 at 24 months, 1.7 ± 1.2 at 36 months, and 1.8 ± 1.3 at 60 months after surgery (*p* < 0.001, respectively). The mean BCVA significantly improved from postoperative 2 weeks. Although 71 (52.6%) eyes had postoperative complications, the cumulative probability of surgical success was 72.6% at 12 months, 66.7% at 24 months, and 63.7% at 36 and 60 months. According to the glaucoma type, the success rate of AGV implantation was more than 50% even after 60 months follow-up, except subgroup of neovascular glaucoma (NVG) due to retinal vein occlusion (RVO).

**Conclusions:**

AGV implantation in patients with refractory glaucoma was, after long-term follow-up, successful overall. Therefore, AGV implantation can be an effective surgical option to reduce the IOP and number of antiglaucoma medications and to improve visual acuity for various glaucoma types.

## Background

Refractory glaucoma is a condition of uncontrolled intraocular pressure (IOP) with deterioration of the optic nerve and visual field despite maximal use of antiglaucoma medications. Neovascular glaucoma (NVG) is the most common type, and various glaucoma types are associated with low success rates after conventional glaucoma filtration surgery under conditions such as aphakia, uveitis, and post-cornea-transplantation trauma [1–6]. Therefore, glaucoma drainage devices have emerged as an effective component of the armamentarium available for the treatment of refractory cases.

The Ahmed glaucoma valve (AGV) is an implanted restrictive drainage valve consisting of thin silicone elastomer membranes that open and close according to IOP variation, thus effectively reducing the incidence of postoperative hypotony [7–9].

To the best of our knowledge, long-term outcomes according to the type of glaucoma in refractory glaucoma have not been previously reported. Therefore, the objective of our study was to evaluate the clinical outcomes and to determine the long-term surgical success rate after AGV implantation in cases of refractory glaucoma according to glaucoma types.

## Methods

Medical records were retrospectively reviewed after approval was granted by the Institutional Review Board of Kyungpook National University Hospital (IRB No. 2020-08-018-001). The requirement for informed consent was waived given the retrospective nature of the study. The review was conducted in accordance with the tenets of the Declaration of Helsinki.

### Study subjects

The study included patients with refractory glaucoma, i.e., the IOP could not be controlled after conventional filtering surgery or administration of antiglaucoma medications, which had undergone AGV implantation (model FP7 with surface area of 184 mm^2^, New World Medical Inc., Rancho Cucamonga, CA, USA) and completed at least 12 months of follow-up period from January 2002 to December 2018.

Patients had a history of intraocular surgery, such as trabeculectomy, cataract surgery, or pars plana vitrectomy, were also included. However, any of the initially reviewed patients with silicone oil filled-eye who had undergone both pars plana vitrectomy and silicone oil tamponade surgery were excluded.

Ophthalmic examinations including the initial best-corrected visual acuity (BCVA) using a Snellen chart, IOP measurement by Goldmann applanation tonometry, slit-lamp examination, and fundus examination were performed. All examinations were repeated at 1 and 2 weeks and at 1, 3, 6, 12, 24, 36 and 60 months after AGV implantation. BCVA values were converted to the logarithm of the minimum angle of resolution (logMAR) scale for statistical analyses.

The type of refractory glaucoma and the number of topical antiglaucoma medications administered before surgery were investigated, as were the changes in the number of medications during the postoperative period. The surgical success rate and postoperative complications were also analyzed by glaucoma type. Finally, for comparative purposes, parameters including IOP, BCVA, and number of antiglaucoma medications were also analyzed.

### Surgical technique

Two surgeons (JPS and DWK) performed all the surgeries, but there was no significant difference in the surgical procedure between surgeons: The AGV implantation procedure was performed under retrobulbar or peribulbar anesthesia. The conjunctival incision was made posteriorly by blunt dissection in the superotemporal quadrant, and a fornix-based conjunctival flap was created in all cases. The valve implant was irrigated with 2 mL of balanced saline solution (Alcon, Fort Worth, USA) using a 27-gauge cannula through the tubing to open the valve mechanism [7]. The plate of the valve was inserted between the superior and lateral rectus muscles, and then joined to the sclera with 7-0 prolene sutures at least 8 mm posterior to the limbus. The drainage tube was trimmed with the bevel facing up and was placed in the anterior chamber through a 23-gauge needle track to allow 2 mm proximity to the limbus. The needle track was anterior and parallel to the plane of the iris. The drainage tube was ligated using a temporary 5-0 prolene intraluminal stent and 8-0 vicryl ligature to prevent early postoperative hypotony and was then inserted carefully so as not to contact the iris or corneal endothelium. The drainage tube was covered with a donor scleral flap of approximately 4 × 4 mm^2^ in size and secured at the four corners of the sclera with 10-0 nylon sutures. The conjunctiva and Tenon’s capsule layer were anchored to the limbus with 8-0 vicryl sutures. No adjunctive antimetabolites (e.g., mitomycin C or 5-Fluorouracil) were used in surgery.

### Successful treatment

The surgery was considered qualified success according to definition of World Glaucoma Association; if the BCVA was better than light perception, and the IOP was 5 – 21 mmHg with medications without serious postoperative complications. Surgical failure was defined as elevation of IOP > 22 mmHg despite maximal tolerated medical therapy or < 5 mmHg after two consecutive visits, BCVA with no light perception, requiring additional surgery for IOP control, or serious postoperative complications such as phthisis bulbi or endophthalmitis.

### Statistical analyses

Statistical analyses were performed using the Statistical Package for the Social Science software version 20 (IBM Corp., Armonk, NY, USA). Wilcoxon-signed rank test was used to compare to compare the mean changes in IOP, number of antiglaucoma medications, and logMAR BCVA from the baseline through the follow-up for all patients and according to the type of glaucoma. The Kaplan–Meier analysis was used to reveal the cumulative probability of the surgical success of AGV implantation; *p*-value < 0.05 was considered significant for all statistical tests.

## Results

A total of 135 patients (135 eyes) were enrolled in this study. Among them, 73 eyes had NVG due to proliferative diabetic retinopathy (PDR), 12 had NVG due to retinal vein occlusion (RVO), and 9 had NVG due to ocular ischemic syndrome (OIS). Of the remaining eyes, 22 had uveitic glaucoma (UG), 9 had primary open-angle glaucoma (POAG), 4 had chronic angle-closure glaucoma (CACG), 4 had pigmentary glaucoma (PG), and 2 had pseudoexfoliation glaucoma (PXG). Among the enrolled patients, 95 were male and 40 were female. The mean age of all patients was 54.3 ± 13.7 years. As for lens status, 40 had phakic eyes, 88 had pseudophakic eyes, and 7 had aphakic eyes. The mean baseline IOP was 35.2 ± 12.0 mmHg, and the mean number of antiglaucoma medications before AGV implantation was 3.4 ± 1.3. BCVA before AGV implantation was logMAR 1.74 ± 1.12, and the mean postoperative follow-up duration was 40.6 ± 25.2 months. The demographic and clinical characteristics of all patients according to the type of refractory glaucoma are summarized in Table 1.

**Table 1:** Demographic and clinical characteristics of the enrolled patients

Characteristics	Type of glaucoma								Total
	NVG PDR	NVG RVO	NVG OIS	UG	POAG	CACG	PG	PXG	
Number of eyes, n	73	12	9	22	9	4	4	2	135
Sex, n									
Male	48	7	7	16	9	3	3	2	95
Female	25	5	2	6	0	1	1	0	40
Age, years	49.6 ± 10.8	59.6 ± 15.9	56.3 ± 11.8	47.7±18.0	57.9 ± 8.0	62.8 ± 9.5	57.5 ± 16.0	52.0 ± 9.9	51.8 ± 13.2
Lens status, n									
Phakia	1	8	6	17	4	2	1	1	40
Pseudophakia	69	4	3	3	4	1	3	1	88
Aphakia	3	0	0	2	1	1	0	0	7
IOP, mmHg	39.3 ± 5.6	37.8 ± 6.3	34.0 ± 5.6	30.8 ± 5.3	25.2 ± 2.9	29.8 ± 3.9	27.0 ± 7.1	29.8 ± 6.6	35.7 ± 11.7
Antiglaucoma medications, n	4.4 ± 0.6	2.7 ± 0.7	2.3 ± 0.7	3.0 ± 0.3	3.0 ± 0.8	3.8 ± 0.3	3.5 ± 0.4	3.5 ± 0.5	3.7 ± 1.3
BCVA, LogMAR	1.93 ± 0.95	2.92 ± 1.32	2.05 ± 1.26	1.24 ± 1.23	0.73 ± 1.01	0.72 ± 0.88	0.70 ± 0.95	0.26 ± 0.06	1.74 ± 1.12
Follow-up, months	41.7 ± 24.7	22.2 ± 26.6	44.8 ± 24.6	38.7 ± 25.6	56.3 ± 11.0	32.3 ± 32.1	60.0 ± 0.0	31.0 ± 41.0	40.6 ± 25.2

Values are presented as the mean ± standard deviations —*CACG* chronic angle closure glaucoma

*IOP* intraocular pressure, *logMAR* logarithm of the minimum angle of resolution, *NVG* neovascular glaucoma, *OIS* ocular ischemic syndrome, *PDR* proliferative diabetic retinopathy, *PG* pigmentary glaucoma, *POAG* primary open angle glaucoma, *PXG* pseudoexfoliation glaucoma, *RVO* retinal vein occlusion, *UG* uveitic glaucoma

### IOP

**Fig. 1:**
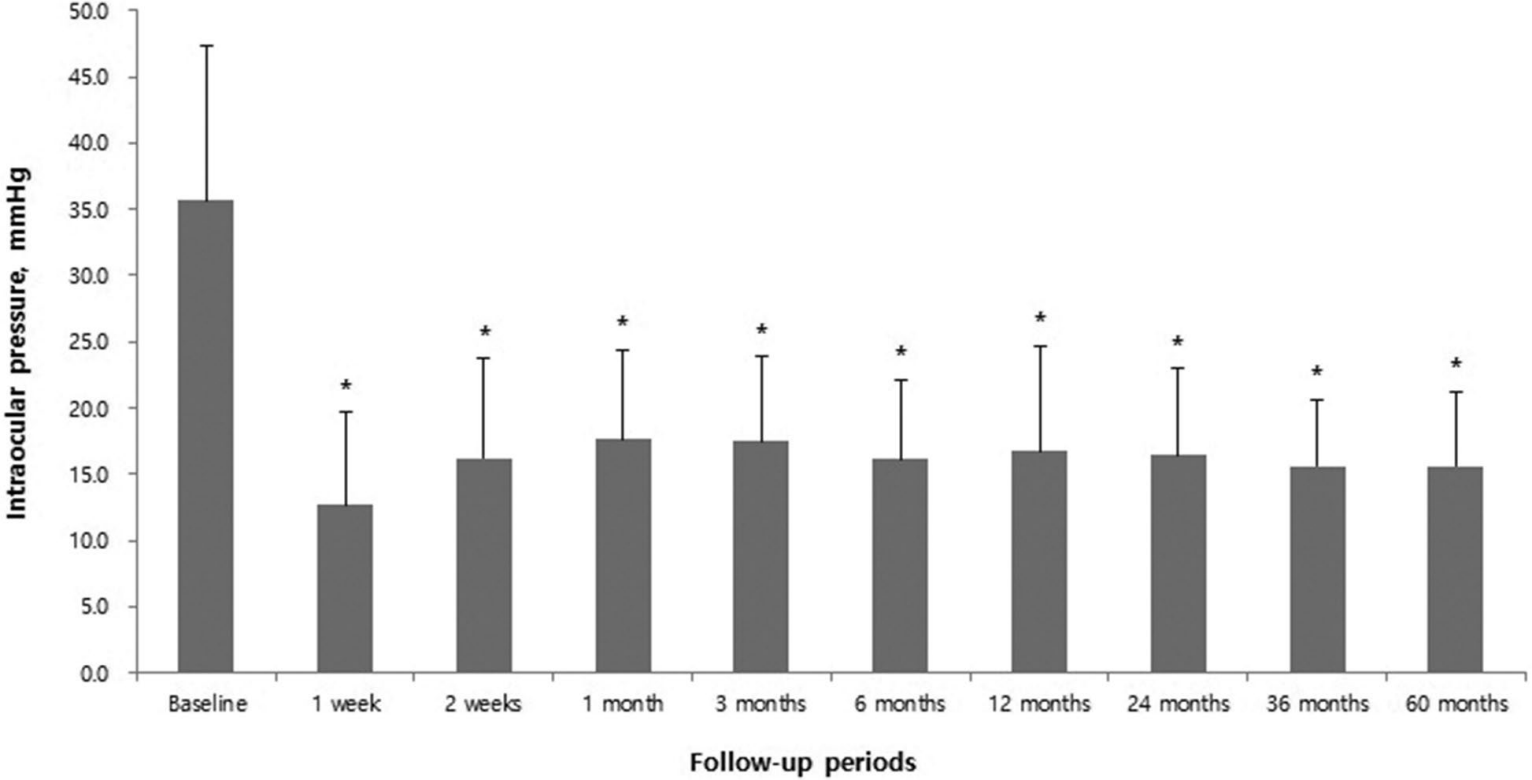
The mean IOP changes during follow-up, which was significantly reduced after AGV implantation. (*: *p* < 0.001 compared to the baseline, respectively)

The mean IOP for all patients was 35.7 ± 11.7 mmHg at baseline, which was significantly reduced to 12.7 ± 7.0 mmHg at 1 week following AGV implantation. The subsequent mean IOP readings were as follows: 16.2 ± 7.5 mmHg at 2 weeks, 17.6 ± 6.8 mmHg at 1 month, 17.5 ± 6.4 mmHg at 3 months, 16.1 ± 6.0 mmHg at 6 months, 16.7 ± 8.0 mmHg at 12 months, 16.4 ± 6.6 mmHg at 24 months, 15.6 ± 5.0 mmHg at 36 months, and 15.6 ± 5.6 mmHg at 60 months (*p* < 0.001, respectively) (Fig. 1). All postoperative IOP changes according to glaucoma type are summarized in Table 2.

**Table 2:** Mean IOP changes according to the type of glaucoma after Ahmed glaucoma valve implantation

IOP, mmHg	Type of glaucoma (n)								Total
	NVG PDR	NVG RVO	NVG OIS	UG	POAG	CACG	PG	PXG	
1 week	13.6 ± 2.7^*^ (73)	14.9 ± 6.2^*^ (12)	11.1 ± 3.0^*^ (9)	10.9 ± 4.4^*^ (22)	11.3 ± 4.3^*^ (9)	11.5 ± 2.6 (4)	11.0 ± 2.1 (4)	9.8 ± 2.4 (2)	12.7 ± 7.0^*^ (135)
2 weeks	16.3 ± 2.6^*^ (73)	15.8 ± 4.3^*^ (12)	15.3 ± 2.9^*^ (9)	15.0 ± 6.1^*^ (22)	17.9 ± 2.2^*^ (9)	18.3 ± 7.2 (4)	19.5 ± 3.9 (4)	16.5 ± 6.1 (2)	16.2 ± 7.5^*^ (135)
1 month	17.9 ± 2.9^*^ (73)	18.6 ± 4.3^*^ (12)	18.6 ± 8.0^*^ (9)	17.5 ± 2.4^*^ (22)	15.2 ± 2.8^*^ (9)	17.8 ± 1.4 (4)	17.5 ± 2.5 (4)	13.5 ± 1.8 (2)	17.6 ± 6.8^*^ (135)
3 months	17.9 ± 6.2^*^ (73)	18.5 ± 3.6^*^ (12)	15.9 ± 4.2^*^ (9)	18.5 ± 3.0^*^ (22)	14.3 ± 1.4^*^ (9)	18.8 ± 3.6 (4)	19.5 ± 6.0 (4)	11.5 ± 3.1 (2)	17.5 ± 6.4^*^ (135)
6 months	16.9 ± 4.8^*^ (73)	18.5 ± 6.6^*^ (12)	13.9 ± 2.6^*^ (9)	14.4 ± 2.6^*^ (22)	14.6 ± 0.8^*^ (9)	18.0 ± 2.3 (4)	15.5 ± 0.4 (4)	11.5 ± 2.6 (2)	16.1 ± 6.0^*^ (135)
12 months	17.5 ± 6.9^*^ (73)	17.9 ± 7.2^*^ (12)	16.8 ± 4.3^*^ (9)	15.8 ± 4.6^*^ (22)	13.4 ± 2.5^*^ (9)	12.5 ± 1.8 (4)	13.5 ± 1.1 (4)	14.5 ± 3.4 (2)	16.7 ± 8.0^*^ (135)
24 months	16.8 ± 5.7^*^ (56)	23.6 ± 6.3^*^ (7)	13.7 ± 2.8^*^ (7)	15.0 ± 3.2^*^ (17)	13.0 ± 1.7^*^ (7)	15.3 ± 4.1 (2)	14.3 ± 2.4 (4)	15.0 ± 0.0 (1)	16.4 ± 6.6^*^ (101)
36 months	16.3 ± 5.3^*^ (41)	20.0 ± 2.9^*^ (4)	18.5 ± 3.2 (6)	13.4 ± 1.5^*^ (13)	12.3 ± 2.0^*^ (4)	13.0 ± 2.3 (2)	12.5 ± 0.4 (4)	18.0 ± 0.0 (1)	15.6 ± 5.0^*^ (75)
60 months	15.5 ± 3.7^*^ (31)	24.3 ± 3.9 (4)	14.0 ± 0.0 (6)	13.6 ± 3.1^*^ (8)	14.0 ± 2.8 (3)	16.0 ± 5.6 (2)	14.0 ± 0.0 (2)	16.0 ± 0.0 (1)	15.6 ± 5.6^*^ (57)

Values are presented as the mean ± standard deviations. *: *p* < 0.05

*CACG* chronic angle closure glaucoma; *IOP* intraocular pressure; *NVG* neovascular glaucoma; *OIS* ocular ischemic syndrome; *PDR* proliferative diabetic retinopathy;*PG*: pigmentary glaucoma; *POAG*: primary open angle glaucoma; *PXG* pseudoexfoliation glaucoma; *RVO* retinal vein occlusion; *UG* uveitic glaucoma

### Number of antiglaucoma medications

**Fig. 2:**
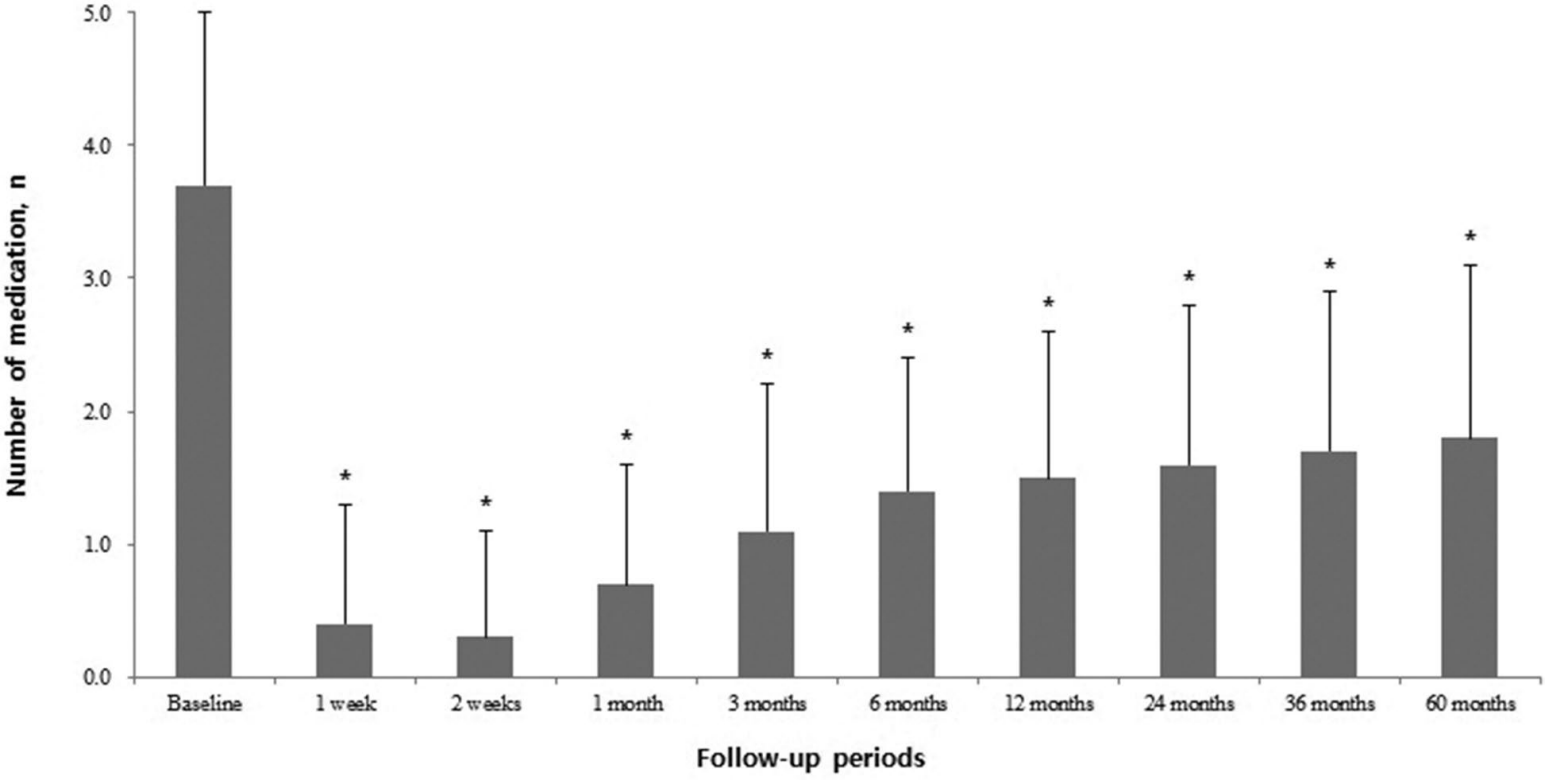
The mean changes of number of antiglaucoma medication during follow-up, which was significantly decreased after AGV implantation. (*: *p* < 0.001 compared to the baseline, respectively)

The mean number of antiglaucoma medications for all patients was 3.7 ± 1.3 at baseline, which was significantly decreased to 0.4 ± 0.9 at postoperative 1 week. The subsequent mean medication numbers were as follows: 0.3 ± 0.8 at 2 weeks, 0.7 ± 0.9 at 1 month, 1.1 ± 1.1 at 3 months, 1.4 ± 1.0 at 6 months, 1.5 ± 1.1 at 12 months, 1.6 ± 1.2 at 24 months, 1.7 ± 1.2 at 36 months, and 1.8 ± 1.3 at 60 months (*p* < 0.001, respectively) (Figure 2). All of the mean postoperative changes in the number of antiglaucoma medications according to glaucoma type are summarized in Table 3.

**Table 3:** Mean changes in the number of antiglaucoma medications according to the type of glaucoma after Ahmed glaucoma valve implantation

Medications, n	Type of glaucoma (n)								Total
	NVG PDR	NVG RVO	NVG OIS	UG	POAG	CACG	PG	PXG	
1 week	0.2 ± 0.4^*^ (73)	0.9 ± 0.7^*^ (12)	0.1 ± 0.2^*^ (9)	0.5 ± 0.6^*^ (22)	0.3 ± 0.5^*^ (9)	0.3 ± 0.3 (4)	0.0 ± 0.0 (4)	1.5 ± 1.1 (2)	0.4 ± 0.9^*^ (135)
2 weeks	0.3 ± 0.6^*^ (73)	0.8 ± 0.5^*^ (12)	0.1 ± 0.2^*^ (9)	0.4 ± 0.4^*^ (22)	0.1 ± 0.2^*^ (9)	0.3 ± 0.3 (4)	0.0 ± 0.0 (4)	2.0 ± 1.4 (2)	0.3 ± 0.8^*^ (135)
1 month	0.7 ± 0.8^*^ (73)	1.1 ± 0.6^*^ (12)	0.1 ± 0.2^*^ (9)	0.5 ± 0.4^*^ (22)	1.0 ± 0.4^*^ (9)	1.0 ± 0.4 (4)	0.8 ± 0.8 (4)	1.5 ± 1.1 (2)	0.7 ± 0.9^*^ (135)
3 months	1.2 ± 0.5^*^ (73)	1.4 ± 0.5^*^ (12)	0.8 ± 0.5 (9)	0.7 ± 0.5^*^ (22)	1.6 ± 0.7^*^ (9)	2.0 ± 0.4 (4)	0.8 ± 0.8 (4)	1.5 ± 0.4 (2)	1.1 ± 1.1^*^ (135)
6 months	1.4 ± 0.5^*^ (73)	1.4 ± 0.5^*^ (12)	1.2 ± 0.5 (9)	0.9 ± 0.4^*^ (22)	2.1 ± 0.5 (9)	1.3 ± 0.5 (4)	1.0 ± 0.7 (4)	2.5 ± 0.4 (2)	1.4 ± 1.0^*^ (135)
12 months	1.5 ± 0.5^*^ (73)	1.4 ± 0.6^*^ (12)	1.3 ± 0.6 (9)	1.0 ± 0.5^*^ (22)	2.1 ± 0.5 (9)	2.0 ± 0.7 (4)	1.8 ± 0.8 (4)	2.0 ± 0.0 (2)	1.5 ± 1.1^*^ (135)
24 months	1.8 ± 0.6^*^ (56)	1.8 ± 0.6 (7)	1.7 ± 0.6 (7)	0.9 ± 0.5^*^ (17)	2.3 ± 0.5 (7)	2.0 ± 0.7 (2)	1.0 ± 0.5 (4)	2.0 ± 0.0 (1)	1.6 ± 1.2^*^ (101)
36 months	1.9 ± 0.6^*^ (41)	1.8 ± 0.7 (4)	3.0 ± 0.0 (6)	0.9 ± 0.5^*^ (13)	2.5 ± 0.5 (4)	1.7 ± 1.0 (2)	1.0 ± 0.7 (4)	2.0 ± 0.0 (1)	1.7 ± 1.2^*^ (75)
60 months	2.1 ± 0.5^*^ (31)	1.7 ± 0.6 (4)	3.0 ± 0.0 (6)	0.7 ± 0.5^*^ (8)	3.0 ± 0.7 (3)	2.0 ± 1.0 (2)	1.0 ± 0.0 (2)	2.0 ± 0.0 (1)	1.8 ± 1.3^*^ (57)

Values are presented as the mean ± standard deviations. *: *p* < 0.05

*CACG* chronic angle closure glaucoma; *IOP* intraocular pressure; *NVG* neovascular glaucoma; *OIS*: ocular ischemic syndrome; *PDR* proliferative diabetic retinopathy;*PG* pigmentary glaucoma; *POAG* primary open angle glaucoma; *PXG*: pseudoexfoliation glaucoma; *RVO* retinal vein occlusion; *UG* uveitic glaucoma

### BCVA

The mean logMAR BCVA of all patients was 1.74 ± 1.12 at baseline, which was improved to 1.72 ± 1.18 at 1 week, 1.59 ± 1.08 at 2 weeks, 1.54 ± 1.08 at 1 month, 1.49 ± 1.13 at 3 months, 1.53 ± 1.20 at 6 months, 1.50 ± 1.19 at 12 months, 1.37 ± 1.20 at 24 months, 1.23 ± 1.16 at 36 months, and 1.00 ± 0.81 at 60 months after AGV implantation. Notably, a significant visual improvement was noted 2 weeks after surgery (*p* < 0.05), which may be due to the disappearance of preoperative corneal edema.

### Postoperative complications

Table 4 presents the postoperative complications by glaucoma type. Early postoperative hypotony occurs in the first 1 to 2 weeks after surgery and is defined as an IOP less than or equal to 6 mmHg [10]. Overall, 79 eyes (58.5%) had postoperative complications: 41 (30.4%) had hyphema, 22 (16.3%) had early-postoperative hypotony, 7 (5.2%) had shallow anterior chamber and 4 (3.0%) eventually developed phthisis bulbi. There was no case of postoperative endophthalmitis, however, 5 (3.7%) occurred tube exposure after surgery and received additional donor scleral patch graft to prevent postoperative endophthalmitis. Postoperative hyphema was the most frequent complication in patients with NVG caused by PDR. However, early-postoperative hypotony was the most frequent complication in patients with NVG due to RVO, NVG due to OIS, UG and POAG.

**Table 4:** Postoperative complications according to the type of glaucoma after Ahmed glaucoma valve implantation.

Postoperative complications, n (%)	Type of glaucoma								Total
	NVG PDR	NVG RVO	NVG OIS	UG	POAG	CACG	PG	PXG	
Hyphema	37	1	1	2	0	0	0	0	41
	(50.7%)	(8.3%)	(11.1%)	(9.1%)	(0.0%)	(0.0%)	(0.0%)	(0.0%)	(30.4%)
Early postoperative hypotony	7	2	3	6	4	0	0	0	22
	(9.6%)	(16.6%)	(33.3%)	(27.3%)	(44.4%)	(0.0%)	(0.0%)	(0.0%)	(16.3%)
Shallow Anterior chamber	1	1	2	1	0	1	1	0	7
	(1.4%)	(8.3%)	(22.2%)	(4.5%)	(0.0%)	(25.0%)	(25.0%)	(0.0%)	(5.2%)
Phthisis bulbi	0	3	0	1	0	0	0	0	4
	(0.0%)	(24.9%)	(0.0%)	(4.5%)	(0.0%)	(0.0%)	(0.0%)	(0.0%)	(3.0%)
Tube exposure	4	0	0	0	0	0	0	1	5
	(5.5%)	(0.0%)	(0.0%)	(0.0%)	(0.0%)	(0.0%)	(0.0%)	(50.0%)	(3.7%)
Total	49	7	6	10	4	1	1	1	79
	(67.1%)	(58.3%)	(66.7%)	(45.5%)	(44.4%)	(25.0%)	(25.0%)	(50.0%)	(58.5%)

*CACG* chronic angle closure glaucoma, *IOP* intraocular pressure, *NVG* neovascular glaucoma, *OIS* ocular ischemic syndrome, *PDR* proliferative diabetic retinopathy, *PG* pigmentary glaucoma, *POAG* primary open angle glaucoma, *PXG* pseudoexfoliation glaucoma, *RVO* retinal vein occlusion, *UG* uveitic glaucoma

### Surgical success rate of AGV Implantation

**Fig. 3:**
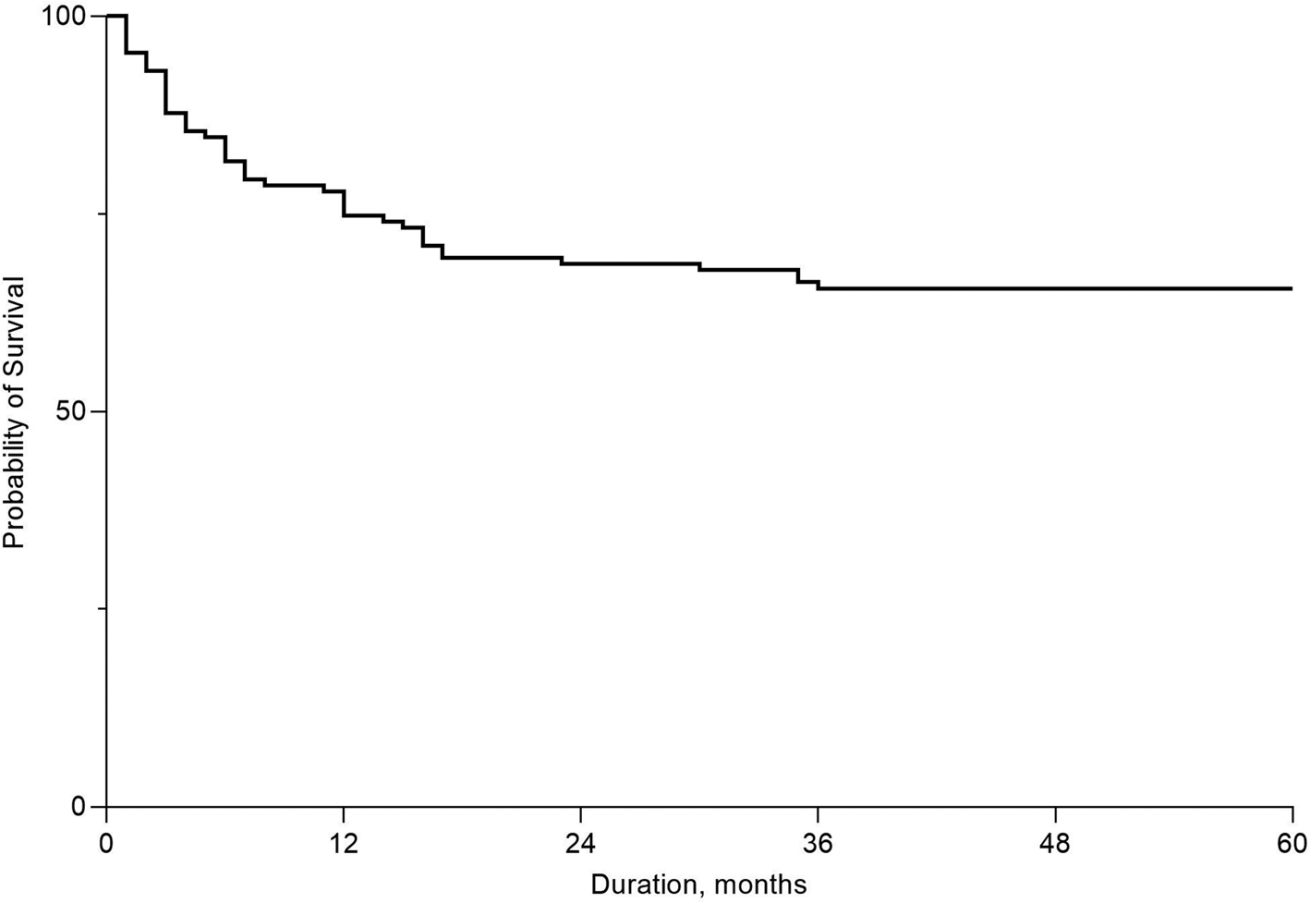
Kaplan–Meier graph illustrating the overall mean cumulative probability of success rate following Ahmed glaucoma valve implantation

**Fig. 4:**
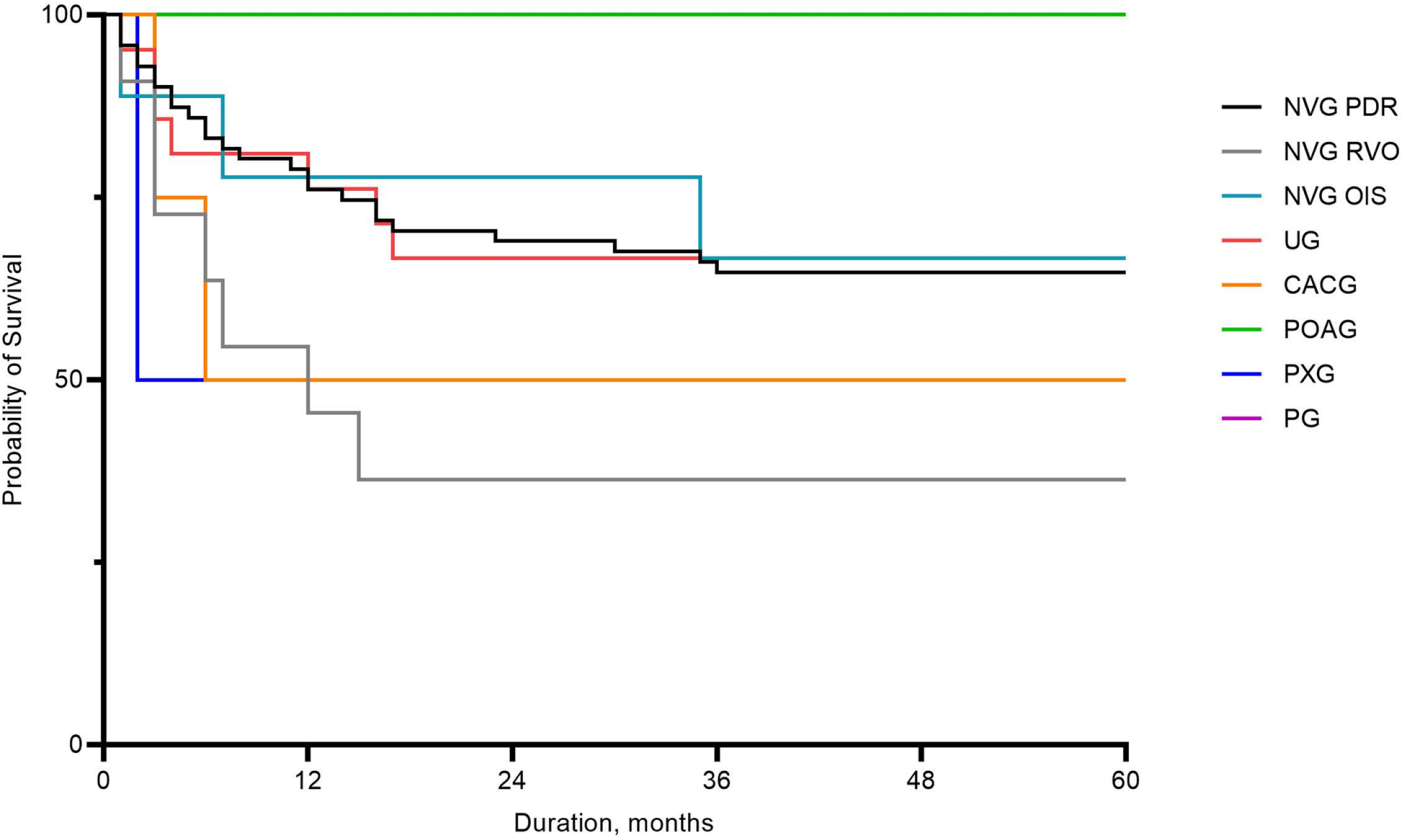
Cumulative probability of success rate according to the subgroup type of refractory glaucoma following Ahmed glaucoma valve implantation; CACG: chronic angle closure glaucoma; NVG: neovascular glaucoma; OIS: ocular ischemic syndrome; PDR: proliferative diabetic retinopathy; PG: pigmentary glaucoma; POAG: primary open angle glaucoma; PXG: pseudoexfoliation glaucoma; RVO: retinal vein occlusion; UG: uveitic glaucoma

In Fig. 3, the Kaplan–Meier plot, shows the overall mean cumulative probability of AGV implantation success. As indicated, the surgical success rate was 72.6% at 12 months, 66.7% at 24 months, and 63.7% at 36 and 60 months. Additionally, Fig. 4 plots the cumulative probability of success by glaucoma type. In NVG due to PDR, the surgical success rate was 76.7% at 12 months, 67.1% at 24 months, and 64.3% at 36 months, 62.9% at 60 months. In NVG due to RVO, the surgical success rate was 50.0% at 12 months, 33.3% at 24, 36, and 60 months. In NVG due to OIS, the surgical success rate was 77.8% at 12 and 24 months, 66.7% at 36 and 60 months. The surgical success rate of UG was 77.3% at 12 months, 63.6% at 24, 36, and 60 months. In CACG and PXG, the surgical success rate was 50.0% after 12 months during the follow-up period. The surgical success rate was 100.0% during the follow-up period in POAG and PG. The success rate of AGV implantation was more than 50% even after 5 years follow-up, except subgroup of NVG due to RVO.

## Discussions

Recently, two studies have reported the results of 5 years of AGV implantation comparing Baerveldt implantation [11, 12]. In the present study, we analyzed the surgical success rate for 60 months and noted results similar to those of relevant previous reports. In addition, we also analyzed the cumulative success rate for 60 months according to each type of glaucoma.

Various glaucoma drainage devices (e.g., Krupin, Ahmed, and Baerveldt) have been employed for the management of refractory glaucoma. The AGV, developed by Mateen Ahmed, provides a more complex mechanism for the control of aqueous outflow. Its valve mechanism consists of thin silicone elastomer membranes that form a Venturi-shaped chamber to respond to IOP variation within the 8 – 12 mmHg range [7].

Although the AGV was designed to prevent postoperative hypotony, it is sometimes prone to malfunction, leakage around the drainage tube, and side effects such as reduced production of aqueous humor [13]. Therefore, making a long tract and/or utilizing a thin 22- or 23-gauge needle for anterior chamber paracentesis and drainage tube insertion have been recommended as additional hypotony-preventive measures [14]. Studies have also reported about intracameral injection of viscoelastics and temporary tube ligation with 8-0 vicryl using a prolene intraluminal stent to prevent excessive filtration immediately after surgery [15, 16].

The incidence rate of postoperative hypotony ranges from 8.8 to 12% for AGVs, to 30% for Krupin–Denver implants, and up to 38% for Baerveldt implants [9, 13, 17–20]. In the present study, the mean incidence of postoperative hypotony was 16.3%, varying from 0 to 44.4% according to the type of glaucoma. In patients with POAG, the incidence rate was 44.4%, which is higher than reports in any other studies; however, this result must be taken with due consideration because the number of patients was relatively small and significance was low. The other reported post-AGV implantation complications include choroidal detachment, hyphema, excessive capsule fibrosis and/or clinical failure, erosion of the tube or plate edge, endophthalmitis, and phthisis bulbi [7, 8, 21, 22].

Postoperative hyphema is also known to be associated with NVG-surgical outcomes [23]. In the present study, the mean incidence of postoperative hyphema was 28.1%, and most frequently arose in patients with NVG due to PDR (46.6%). However, the hyphema was naturally absorbed within 1 or 2 weeks after surgery, and there was no case of drainage tube occlusion due to blood clots in the anterior chamber. On the contrary, patients with other causes of NVG, such as RVO or OIS, had higher incidences of postoperative hypotony complications. We thought that the higher incidence of postoperative hyphema in NVG due to PDR was the results of advanced status of diabetic retinopathy in enrolled patients. Most PDR cases had advanced stages of diabetic retinopathy; essentially, NVG also occurred after PDR treatment despite patients having already received treatments for PDR such as panretinal photocoagulation, intravitreal anti-vascular endothelial growth factor (VEGF) injection, and vitrectomy.

There have been numerous reports on the use of anti-VEGF to reduce neovascularization in the management of NVG [24, 25]; however, the effectiveness of preoperative anti-VEGF treatment for glaucoma surgery remains controversial. In this study, 51 of 94 NVG eyes (54.3%) and 39 of 73 NVG due to PDR eyes (53.4%) received anti-VEGF treatment before AGV implantation; however, no significant correlation was observed in the surgical success rate (*p* = 0.184 in NVG, *p* = 0.194 in NVG due to PDR, Chi-squared test).

The reported surgical success rates of AGV implantation vary. Coleman et al. [7] reported a success rate of 78%, while Nouri-Mahdavi and Caprioli [26] reported 71% at 12 months after surgery. Topouzis et al. [9] reported a cumulative success rate of 87% for 12 months, 82% for 24 months, and 76% for 36 and 48 months after surgery. Lee et al. [27] reported a cumulative success rate of 83.0% after 12 months, 75.8% after 24 months, and 68.2% after 36 months for Korean patients. In this study, the results of surgical success rate for each type of glaucoma were similar to those in other reports on NVG due to PDR and UG. Moreover, the success rate over the course of the follow-up was 100% in POAG and PG cases, much higher than a previously reported rate of 57% with POAG patients 30 months after surgery [28]. However, this comparison must be evaluated with care, as the number of patients with POAG in the present study was relatively small.

The cumulative surgical success rates of 72.7% at 12 months, 68.1% at 24 and 36 months, 54.5% at 48 and 60 months for patients with UG. Glaucoma drainage device is an appropriate primary surgical procedure in particularly in UG, considering complications such as postoperative inflammatory response and excessive fibrosis after trabeculectomy with or without antimetabolites. Reported success rates with AGV implantation in UG patients are 66.6 to 94.4% at 1 year and 60.0 to 66.6% at 2 years [29, 30], which is similar to those of relevant previous reports.

On the contrary, our results revealed relatively lower cumulative success rates of 77.8% at 12 and 24 months and 38.9% at 36 and 60 months for patients with NVG due to OIS. Moreover, the rates were similarly lower for NVG due to RVO: 41.7% at 12 months and 27.8% at 24, 36, and 60 months. Mermoud et al. [28] reported that patients with NVG due to RVO had relatively poor surgical outcomes compared with patients with NVG due to PDR after implantation of Molteno devices. They revealed that surgical failure was caused by visual loss from aggravation of RVO and phthisis bulbi. Similarly, 24.9% of our cases of NVG due to RVO eventually developed phthisis bulbi. In this study, all cases were diagnosed NVG secondary to central retinal vein occlusion. Nowadays, anti-VEGF intravitreal injection therapy was introduced not only to treat retinal edema, but also to reduce neovascular complications due to ischemia in RVO. However, this study included before and after anti-VEGF injection was introduced. Among them, only 2 patients were previously received anti-VEGF therapy. As a result, it is thought that the success rate was relatively low in NVG due to RVO. Given that OIS is caused by ocular hypoperfusion due to stenosis or occlusion of the common or internal carotid arteries, it is known to have a poor prognosis and a correspondingly low success rate, owing specifically to systemic comorbidity despite surgical treatment for NVG [31]. Accordingly, our results present a low cumulative success rate in cases of glaucoma caused by RVO and OIS relative to the other causes of glaucoma.This study has two limitations. First, it is retrospective. Second, there is a slight variation in the numbers of patients with glaucoma according to glaucoma type: some types have relatively limited patient representation compared with others. Nevertheless, the significance of this study lies in its analysis of the long-term (60-month) cumulative surgical outcomes of AGV implantation separately for various types and causes of glaucoma.

## Conclusions

AGV implantation demonstrated a cumulative surgical success rate more than 50% over the course of 60 months of follow-up for most causes of refractory glaucoma, despite differences in success rates by glaucoma type. AGV implantation effectively reduced the IOP and the number of antiglaucoma medications and improved visual acuity. Therefore, AGV implantation can be a sound surgical option for various types of refractory glaucoma, with the caveat that prognostic differences should be recognized among the types and causes of glaucoma.

## Data Availability

The datasets used and/or analyzed during the current study available from the corresponding author on reasonable request.

## Abbreviations

AGV: Ahmed Glaucoma Valve
BCVA: Best Corrected Visual Acuity
CACG: Chronic Angle Closure Glaucoma
IOP: Intraocular Pressure
NVG: Neovascular Glaucoma
OIS: Ocular Ischemic Syndrome
PDR: Proliferative Diabetic Retinopathy
PG: Pigmentary Glaucoma
RVO: Retinal Vein Occlusion
POAG: Primary Open Angle Glaucoma
PXG: Pseudoexfoliation Glaucoma
UG: Uveitic Glaucoma
